# Experience in Intra-arterial Chemotherapy using Two Protocols for the Treatment of OSCC over Two Decades at the University Hospital Vienna

**DOI:** 10.6061/clinics/2018/e433

**Published:** 2018-10-10

**Authors:** Christina Eder-Czembirek, Sascha Rechinger, Gabriela Kornek, Edgar Selzer, Rudolf Seemann

**Affiliations:** I Department of Cranio, Maxillofacial and Oral Surgery, Medical University Vienna, Vienna, Austria; II University Clinic of Dentistry Vienna, Vienna, Austria; III University Clinic of Internal Medicine, Department of Oncology, Medical University Vienna, Vienna, Austria; IV University Clinic of Radiotherapy, Medical University Vienna, Vienna, Austria

**Keywords:** Intra-Arterial Chemotherapy, Oral Squamous Cell Carcinoma, Locally Recurrent, Cisplatin, Methotrexate, Head and Neck

## Abstract

**OBJECTIVES:**

This retrospective study performed a comprehensive analysis of the usage of intra-arterial chemotherapy (iaCh) for locally recurrent UICC stage IV oral squamous cell carcinoma (OSCC) over two decades at the Department of Cranio-Maxillofacial and Oral Surgery at the University Hospital Vienna to assess the utility of its future use.

**METHODS:**

Between 1994 and 2014, iaCh was indicated in 48 OSCC cases. In these, the two most frequent iaCh schemes, cisplatin/5-fluorouracil (Cis/5-FU) and methotrexate/bleomycin (MTX/Bleo), were chosen for further analysis. The effect on survival of two distinct intra-arterial protocols and their covariates were analyzed with the Kaplan-Meier method as well as univariate and multivariate Cox proportional hazard regression models.

**RESULTS:**

The mean follow-up period was 29.91 months. The two intra-arterial chemotherapy groups did not differ significantly in sample size, demographic data or therapeutic covariates. The Cis/5-FU iaCh regimen was associated with significantly better overall survival (median OS 2.6 years *vs*. 1.3 years; *p*=0.002) and had a beneficial effect on survival (HR=3.62, *p*=0.015). Side effects occurred at a frequency similar to that described in the literature for intravenous chemotherapy (ivCh).

**CONCLUSIONS:**

These results suggest a preference for administering Cis/5-FU for iaCh. Nevertheless, due to economic considerations in healthcare expenditures, there is no future for iaCh in the treatment of head and neck carcinomas because ivCh is known to be equivalent.

## INTRODUCTION

The majority of oral squamous cell cancers are detected in locally advanced stages, requiring primary radiotherapy (RT) or neoadjuvant RT in combination with intravenous chemotherapy (ivCh), biotherapy, and surgery 1-7, and patients show encouraging overall survival rates. However, no consensus has been found regarding therapeutic options for recurrent and, especially, unresectable head and neck squamous cell carcinoma (HNSCC) 7.

Even though impressive clinical responses, together with the potential to reduce systemic side effects 8, have been reported, the administration of chemotherapeutics intra-arterially instead of systemically for the treatment of head and neck cancer is neither a routine nor an alternative approach, although it was initially regarded as a therapeutic option for the treatment of unresectable recurrences. On the one hand, this is due to the lack of uniform selection criteria (localization, stage, and histology) and treatment schedules 9-11. On the other hand, randomized studies that compared intra-arterial versus intravenous chemoradiation showed that iaCh was not superior to standard treatment 12-14.

The purpose of this retrospective study was to provide a comprehensive analysis of the usage of iaCh for locally recurrent UICC stage IV oral squamous cell carcinoma (OSCC) over two decades at the Department of Cranio-Maxillofacial and Oral Surgery at the University Hospital Vienna in an attempt to determine its future utility. Cisplatin, the basis of all head and neck chemotherapy, and methotrexate, the second most common iaCh drug, were administered in consistent intra-arterial dual-drug protocols (Cis/5-Fu *vs*. MTX/Bleo).

## METHODS

The retrospective study was approved by the institutional ethics committee (EKNr. 1627/2013).

### Patients

**Figure 1:**
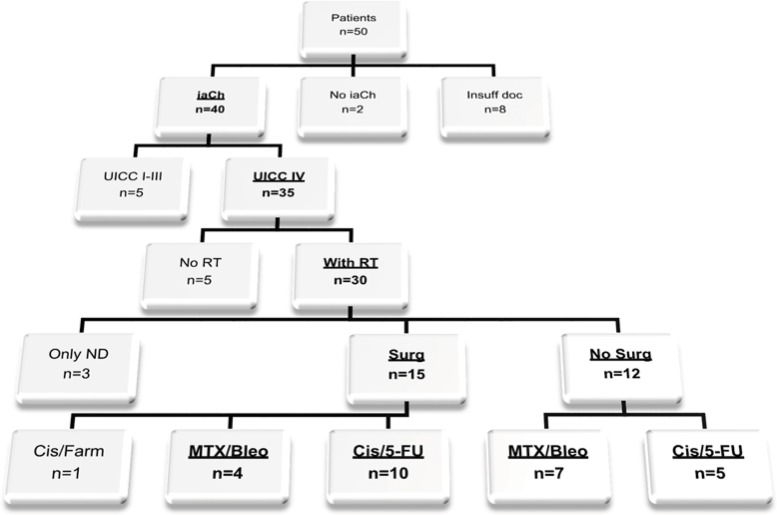
Flow chart for strict selection criteria (top to bottom). Underlined therapies in the bottom line show the final sample of included patients (n=26; insuff doc=insufficient documentation; Cis/Farm, cisplatin=farmorubicin; Surg=surgery; RT=radiotherapy).

The medical records of 50 patients with documented intention for iaCh treatment from January 1994 to December 2014 were searched. To homogenize the collection, the inclusion criteria included locally recurrent UICC stage IV OSCC (TNM Classification of the UICC, 7^th^ edition 2009) and intra-arterial treatment with Cis/5-FU or MTX/Bleo. Radiotherapy, as a standard treatment for advanced head and neck cancer, and administration with curative intention were obligatory in the patient's recent medical history, and thus, no further treatment option existed for relapse. Previous surgical procedures were required to be intended as curative as well. Former ivCh was optional. Patients receiving no or other iaCh agents or those who had never been irradiated for OSCC treatment were excluded from the analysis. To avoid potentially confounding factors, all patients with insufficient documentation were excluded from further analysis (Figure 1).

### Intra-arterial regimens

Generally, iaCh was indicated in cases of former cisplatin ivCh and those with the intention to reduce the systemic dose or the patient's refusal to receive further ivCh because of systemic side effects. The two iaCh therapy regimes consisted of cisplatin and 5-fluorouracil (Cis/5-FU) and methotrexate and bleomycin (MTX/Bleo). Each patient was assigned to one or the other regimen by the recommendation of the prescribing oncologist. The Cis/5-FU protocol comprised 50 mg/m^2^ cisplatin over two hours on day one and day eight and 250 mg/m^2^ 5-fluorouracil over two hours from day one to day fifteen. MTX/Bleo was given as a two-hour infusion (25 mg methotrexate and 15 mg bleomycin) from day one to day twenty. Sodium thiosulfate and calcium folinate were routinely administered only when elevated serum levels of chemotherapy could be expected due to, for example, renal failure, or in the case of reduced fluid intake.

Catheter placement was performed by an anterograde approach directly through the external carotid artery under general anesthesia. Correct catheter placement was routinely checked during the insertion and before each chemotherapy session by intra-arterial application of 0.4% indigo carmine solution. The catheters were heparinized after every manipulation.

### Adverse effects

The adverse effects were classified according to the Common Terminology Criteria for Adverse Events (CTCAE, Version 4.03). Information about mucositis, blood disorders, ischemia and neurologic disorders was obtained from the patients' charts.

### Staging and follow-up

Staging was performed according to the TNM Classification (UICC, 7^th^ edition 2009). All patients had CT or MRI scans of the head and neck region, a chest X-ray or CT-scan, plus sonography of the upper abdomen at first diagnosis and biannually after primary treatment.

### Data acquisition and statistical analysis

The patient- and treatment-specific data were collected from patient charts and internal administration programs of the clinic. The outpatient data were collected from the hospital database management system Clinicware^®^ (Agfa HealthCare, Bonn, Germany). Survival was assessed by obtaining life data from central registers and insurance companies as well as through direct contact with the patient or the patient's relatives. Complete data regarding survival status were available for all analyzed cases. The Pearson's Chi-square test and the Welch two-sample t-test were used to verify the homogeneity of the data in terms of demographic, clinical, pathological and treatment variables.

Overall survival was calculated using the Kaplan-Meier method. The therapeutic effects (i.e., Cis/5-FU *vs*. MTX/Bleo, no surgery *vs*. surgery, no intravenous chemotherapy *vs*. intravenous chemotherapy) were analyzed in uni- and multivariable Cox proportional hazard regression models. The adverse effects of the two iaCh groups were compared using Fisher's exact test. A significance level of α=0.05 was used as the cut-off. The open-source statistical programming environment “R” (version 3.1.1, R Core Team, 2014) was used to calculate survival estimates, to analyze the impact of the variables on survival, and to generate graphs.

## RESULTS

Finally, twenty-six patients with locally recurrent stage IV OSCC were eligible for further analysis (Figure 1). Of these, fifteen patients were treated with Cis/5-FU (58%) compared to eleven patients treated with MTX/Bleo (42%). The two investigated groups did not significantly differ in demographic and clinical parameters or in pathological and treatment covariables (Table 1). All included patients received iaCh and had undergone former radiotherapy for UICC stage IV OSCC. No patient was lost to follow-up. The median follow-up period was 16.89 months (min 1.48 months, max 134.3 months). Twenty patients (77%) had disease-specific causes of death, whereas four patients (15%) died from other causes (pneumonia, dilated cardiomyopathy) and one of an unknown cause.

### Overall survival

**Figure 2:**
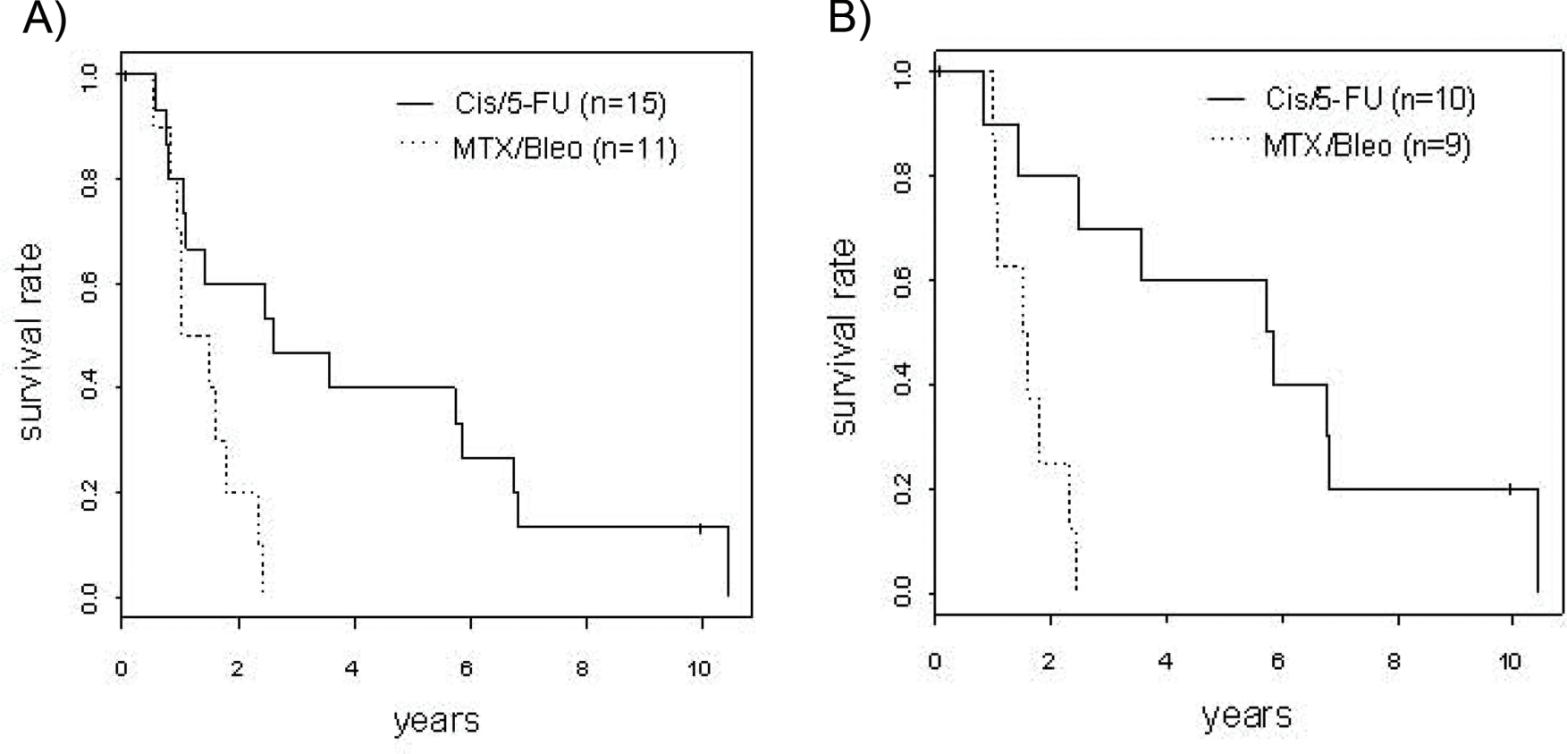
. Kaplan-Meier analysis of overall survival (A) and with the effect of intravenous chemotherapy (B).

The median overall survival (OS) time was 2.6 years in the Cis/5-FU group, which was significantly increased compared to the 1.3 years in the MTX/Bleo group (Figure 2). Correcting for all known confounders, which did not significantly differ between the iaCh groups, the Cis/5-FU group still showed superior survival (Table 2).

After one year, three patients in the Cis/5-FU group (1-year OS=80%; 95% CI 0.62-1.00) and three patients in the MTX/Bleo group (1-year OS=70%; 95% CI 0.47-1.00) had died. After two years, nine patients treated with Cis/5-FU (2-year OS=60%; 95% CI 0.40-0.91) were alive, and three patients treated with MTX/Bleo (2-year OS=20%; 95% CI 0.06-0.69) were alive. Six patients who received Cis/5-FU were alive after five years (5-year OS=40%; 95% CI 0.22-0.74), whereas there were no survivors in the MTX/Bleo group after 2.4 years.

### Adverse effects

The incidence rate for adverse effects due to iaCh treatment was 61.5% (16 of 26 patients) with significantly less adverse effects in the Cis/5-FU group (Fisher's exact test for count data: OR=0, 95% CI 0.0-0.946, *p*=0.024). Twelve patients (46%; Cis/5-FU n=6; MTX/Bleo n=6) suffered from grade 3 mucositis during iaCh treatment. Seven patients (27%; Cis/5-FU n=2; MTX/Bleo n=5) received transfusions because of grade 3 blood disorders (seven cases with red cellconcentrates due to anemia and four cases with platelet concentrates due to thrombocytopenia). Signs of reversible hemiplegia were documented in one patient (Cis/5-FU).

## DISCUSSION

Fifty years of clinical experience in intra-arterial chemotherapy of head and neck cancer has not lead to homogenous patient selection criteria or universally valid treatment schedules and drug administration procedures 10,15-19. Herein, we describe, for the first time, the institutional results of two decades of iaCh treatment of locally recurrent UICC stage IV OSCC. Strict patient selection criteria were applied to create a homogenous patient collective. Cisplatin, the standard agent for squamous cell carcinoma of the head and neck 1,2, and methotrexate, the second most common iaCh drug used internationally for OSCC, were administered in consistent intra-arterial dual-drug protocols (Cis/5-Fu *vs*. MTX/Bleo).

The Cis/5-FU group showed significantly better overall survival and significantly less adverse effects in uni- and multivariable analyses.

A broadly similar sample of patients with UICC stage IV OSCC was presented by Balm et al. 20. These authors reported a 1-year OS of 69% for the oral cavity subgroup (n=20) with single Cis iaCh and multimodal treatment. The presented study cohort showed a 1-year OS of 80% after Cis/5-FU iaCh. Whereas Balm et al. 20 considered salvage surgery only in cases of regional residual disease, the included study patients underwent radical tumor surgery for curative purposes.

As previously mentioned, iaCh with Cis/5-FU caused significantly fewer side effects than MTX/Bleo, yet despite the reduction in the cisplatin dose, the expectation of some authors to decrease the rate and severity 11,21 could not be addressed. Grade 3 toxicities appeared after iaCh (46%) with an incidence similar to that after conventional radiochemotherapy (up to 43%) 22,23, which may have been due to the polychemotherapy protocol.

Because this study was conducted in a retrospective manner, there are some obvious limitations associated with the analysis, such as susceptibility to deficiencies in data recording and collection. Moreover, there was no matching control group for the named period because recurrent UICC stage IV OSCC was applied as a selection criterion. Finally, the study consisted of a final group of 26 patients and therefore may lack the statistical power to show associations. One strength of this study is the completeness of the study cohort, as no patient was excluded due to loss of follow-up or the inability to retrieve medical records.

IaCh with cisplatin as an intra-arterial agent can be considered for consecutive treatment after standard therapy in patients with recurrent oral cancer, with the aim of delivering a higher dose to the tumor 21. The main disadvantage of this approach resides in the feasibility of the protocol, which can be performed at only a few institutes with the infrastructure and resources for this specialized procedure and its elevated costs 9,17,24 compared to conventional systemic chemotherapy. In conclusion, it must be emphasized that chemotherapy is effective for the treatment of advanced and/or recurrent head and neck cancer 25,26, but due to the aforementioned facts, the intravenous route will remain the standard of care for head and neck cancer.

## AUTHOR CONTRIBUTIONS

Eder-Czembirek C was responsible for the data acquisition, manuscript writing and editing. Rechinger S was responsible for the data acquisition. Kornek G was responsible for the manuscript writing and data acquisition. Selzer E and Seemann R were responsible for the manuscript editing and statistical analysis.

## Figures and Tables

**Table 1:** Demographic data of the included patients (n=26).

Characteristics		Cis/5-FU n=15	MTX/Bleo n=11	Statistics
Sex^a^	Male (n=17)	9	8	Chi^2^=0.066
	Female (n=9)	6	3	*p*=ns
Age at diagnosis^b^	Mean (S.E.)	56.5±13.5	55.0±9.7	t=0.350
	Min-Max	24.9-77.2	39.1-70.2	*p*=ns
Localization^a^	Lower Jaw (n=13)	5	8	Chi^2^=4.264
	Upper Jaw (n=5)	4	1	*p*=ns
	Tongue (n=7)	5	2	
	Cheek (n=1)	1	0	
ECOG^c^	0 (n=16)	10	6	W=45.5
	1 (n=6)	4	2	*p*=ns
	3 (n=4)	1	3	
Additional treatments^a^	no ivCh & no surgery (n=4)	2	2	Chi^2^=3.793
	ivCh & no surgery (n=8)	3	5	*p*=ns
	no ivCh & surgery (n=3)	3	0	
	ivCh & surgery (n=11)	7	4	

Abbreviations: Cis/5-FU, cisplatin/5-fluorouracil; MTX/Bleo, methotrexate/bleomycin; ivCh, intravenous chemotherapy; ns, not significant.

^a^Pearson’s Chi-square test; ^b^ Welch two-sample t-test; ^c^ Wilcoxon rank sum test. Data are shown according to the intra-arterial chemotherapy regimens Cis/5-FU and MTX/Bleo as well as the dummy variables intravenous chemotherapy (ivCh) and surgery.

**Table 2:** Uni- and multivariable Cox proportional hazard analyses of iaCh with Cis/5-FU or MTX/Bleo (n=26) and dummy variables such as intravenous chemotherapy (ivCh; 12 times prior to iaCh, seven times subsequently to iaCh) and surgery.

		β	HR (e^β^)	CI	*p*-value
Univariable	MTX/Bleo *vs*. Cis/5-FU	1.29	3.96	1.28-10.25	0.0153
	ivCh *vs*. no ivCh	-1.38	0.25	0.10-0.67	0.006
	surgery *vs*. no surgery	-0.38	0.69	0.30-1.58	ns
Multivariable	MTX/Bleo *vs*. Cis/5-FU	1.95	7.01	2.05-23.86	0.002
	ivCh *vs*. no ivCh	-2.14	0.12	0.04-0.38	0.0003
	surgery *vs*. no surgery	-0.17	0.84	0.34-2.01	ns

Abbreviations: Cis/5-FU, cisplatin/5-fluorouracil; MTX/Bleo, methotrexate/bleomycin; ivCh, intravenous chemotherapy; ns, not significant.
